# Phonological perception by birds: budgerigars can perceive lexical stress

**DOI:** 10.1007/s10071-016-0968-3

**Published:** 2016-02-25

**Authors:** Marisa Hoeschele, W. Tecumseh Fitch

**Affiliations:** Department of Cognitive Biology, Althanstrasse 14, 1090 Vienna, Austria

**Keywords:** Comparative cognition, Acoustic perception, Animal phonology, Metrical stress, Evolution of language, Budgerigars, Operant conditioning

## Abstract

Metrical phonology is the perceptual “strength” in language of some syllables relative to others. The ability to perceive lexical stress is important, as it can help a listener segment speech and distinguish the meaning of words and sentences. Despite this importance, there has been little comparative work on the perception of lexical stress across species. We used a go/no-go operant paradigm to train human participants and budgerigars (*Melopsittacus undulatus*) to distinguish trochaic (stress-initial) from iambic (stress-final) two-syllable nonsense words. Once participants learned the task, we presented both novel nonsense words, and familiar nonsense words that had certain cues removed (e.g., pitch, duration, loudness, or vowel quality) to determine which cues were most important in stress perception. Members of both species learned the task and were then able to generalize to novel exemplars, showing categorical learning rather than rote memorization. Tests using reduced stimuli showed that humans could identify stress patterns with amplitude and pitch alone, but not with only duration or vowel quality. Budgerigars required more than one cue to be present and had trouble if vowel quality or amplitude were missing as cues. The results suggest that stress patterns in human speech can be decoded by other species. Further comparative stress-perception research with more species could help to determine what species characteristics predict this ability. In addition, tests with a variety of stimuli could help to determine how much this ability depends on general pattern learning processes versus vocalization-specific cues.

**Electronic supplementary material:**

The online version of this article (doi:10.1007/s10071-016-0968-3) contains supplementary material, which is available to authorized users.

## Introduction

A vast amount of the information contained in speech is lost in written language, especially when unpunctuated. Nonetheless, the intonation, rhythm and emphasis of words and syllables expressed through unwritten attributes of sound such as pitch, duration and amplitude, play a large role in our interpretation of meaning. These features within speech are collectively referred to as prosody. In the last few years, researchers have begun to study the perception of prosody by non-human animals (Ramus et al. 2000; Toro et al. 2003; Naoi et al. 2012; de la Mora et al. 2013; Spierings and ten Cate 2014). The study of human speech perception by non-human animals helps answer questions about the universality of features such as prosody in communication across the animal kingdom (Kriengwatana et al. 2015).

This surge in recent animal work is a logical next step from cross-cultural human data. Although there is superficial variation in prosodic cues across languages, a deeper look at these cues reveals important similarities. One example of this is metrical phonology, the stress or emphasis of words and syllables, which plays a vital role in the perception of speech and in language acquisition (Cutler and Norris 1988; Cutler 2012). In English, lexical stress alone can change the meaning of a word. For example, the word permit with emphasis on the first syllable is a noun, whereas the word permit with emphasis on the second syllable is a verb. Similarly, in sentences lexical stress can clarify meaning and help segment the speech into words (Cutler et al. 1997; Kuhl 2004). Stressed syllables and words possess altered acoustic parameters relative to other surrounding elements. They are generally higher in pitch, longer in duration, higher in amplitude and include longer and fuller vowels (Fry 1958). Thus, there are multiple acoustic cues that could be used to identify stressed elements, and indeed all of these features may be used by humans when interpreting whether a speech element is stressed (Fry^1958^; Lehiste and Fox 1992; Kohler 2012). Despite considerable variation in the role of stress across languages, metrical patterns in all natural human languages are based on universal organizational principles (Hayes 1995; Lahiri 2001; Cutler 2012).

Not only is metrical phonology highly relevant in language, but these principles appear to be used in other domains as well, such as music (Lerdahl and Jackendoff 1983; Jackendoff 1987; Lerdahl 2001; Jackendoff and Lerdahl 2006; Fabb and Halle 2012; Vaux and Myler 2012). We tend to group elements, both in the visual and acoustic modality, based on simple Gestalt principles such as similarity and proximity. Elements that are more similar to one another, such as close together in pitch, tend to be grouped together. Elements that are closer together in time or space also tend to be grouped together (although auditory grouping is rather weak concerning spatial cues, see Bregman 1990). In music as in speech, louder and longer elements are more likely to be treated as stressed, and changes in pitch also form boundaries between stressed and unstressed elements (Jackendoff and Lerdahl 2006). These groupings allow us to parse the underlying stress patterns. Importantly, there are often several possible underlying stress patterns that can emerge from continuous streams of acoustic information. The simplest example is distinguishing whether a continuous pattern of alternating stressed and unstressed elements is heard as a trochaic (stressed followed by unstressed) or an iambic (unstressed followed by stressed) pattern.

The iambic–trochaic law (Bolton 1894) was proposed to summarize what occurs perceptually when humans are confronted with a continuous acoustic stream of two alternating elements. If the two elements differ only in intensity or frequency, we will hear a trochaic pattern, i.e., we perceive the more stressed (higher intensity/frequency) element as coming “first” in each pair. If the two elements differ only in duration, we will hear an iambic pattern, i.e., we perceive the more stressed (longer) element as coming “second” in each pair (Bolton 1894). Despite stress being more important in some languages than others, similar grouping based on intensity seems to occur cross-culturally (Hay and Diehl 2007; Iversen et al. 2008). However, grouping based on duration is not found in Japanese speakers, and is not yet present in 7-month-old infants (Iversen et al. 2008; Bion et al. 2011). A similar difference between groupings based on intensity and duration was recently found in rats (*Rattus norvegicus*; de la Mora et al. 2013). The rats also grouped stimuli consistently based on intensity, but not duration. This suggests that the iambic–trochaic law may be based in fundamental perceptual grouping mechanisms found across species. Further support for this comes from the fact that the iambic–trochaic law appears to also apply to visual stimuli (Peña et al. 2011), which suggests that these grouping mechanisms are not specific to language and music.

Surprisingly, comparative research on animal language processing has tended to focus on syntax rather than prosodic aspects (such as metrical phonology) of language (e.g., Hauser et al. 2002; Fitch and Hauser 2004; Patel 2003; Pinker and Jackendoff 2005; Gentner et al. 2006; Stobbe et al. 2012; ten Cate and Okanoya 2012). However, as Yip (2006) points out, the debate between researchers on what aspects of language can be found in non-human animals could be greatly enhanced through studies of phonology. This point is becoming increasingly clear with results such as those found in the rats that also showed similar iambic/trochaic grouping patterns to those found in infants (de la Mora et al. 2013). Recent evidence shows that java sparrows (*Lonchura oryzivora*) and zebra finches (*Taeniopygia guttata*), both vocal learning songbirds (that is, species that learn to produce their species-specific vocalizations based on perceptual input from conspecifics; Farabaugh et al. 1994; see Tyack 2008 for a review on vocal learning), can attend to prosodic cues in human speech (Naoi et al. 2012; Spierings and ten Cate 2014). Not only this, but the work on zebra finches showed that although the finches attend to both syntactic and prosodic cues in human speech, they attend primarily to prosody when discriminating vocalizations (Spierings and ten Cate 2014). We were interested to know whether an animal can rely on stress pattern alone to categorize words, i.e., can an animal distinguish between trochaic and iambic stress when presented with two-syllable words?

Here, we look at the perception of metrical stress in humans and a common pet parrot species: the budgerigar (*Melopsittacus undulatus*). Budgerigars are an ideal species for comparative work of metrical stress. They are a small Australian parrot species that are easy to handle and train. Budgerigars are not only vocal learners, but they are also vocal mimics, i.e., they can learn to reproduce sounds that occur in their environment but are not species-specific (Gramza 1970). In addition, budgerigars have shown to be able to synchronize to a beat (Hasegawa et al. 2011), have highly accurate pitch perception (Weisman et al. 2004), can detect complex harmonic changes (Lohr and Dooling 1998), and have been shown to be able to discriminate human vowels (Dooling and Brown 1990). Thus, they have been shown to attend in detail to all potential cues of metrical stress, and to reproduce and move to the sounds they hear in their environment.

We conducted a go/no-go operant training procedure to compare humans and budgerigars in their ability to discriminate stress patterns in two-syllable nonsense words. After training, members of both species were tested with novel exemplars to see whether they had learned a rule or used rote memory to solve the task. We then tested each species with stimuli with some cues removed to attempt to pinpoint what features of lexical stress are most relevant for each species.

## Materials and methods

### Participants

Thirty-one adult humans participated in the experiment (13 males, 18 females) at the University of Vienna. They were recruited either directly by a research assistant or through an online system (SONA) where potential participants were registered and could sign up for experiments for monetary compensation. Most of the pool registered with SONA was made up of students recruited through advertisements around the university. None of the participants had any prior knowledge about the experiment. Informed consent was obtained from all individual participants included in the study.

Six budgerigars participated in the task (5 males, 1 female). All were roughly 8 months old when they began the experiment and were experimentally naïve. All 6 birds were housed together in an aviary (2 × 1 × 2 m). Birds were trained 5 days a week. At all times, birds had free access to water in the aviary. Food pellets (Avifood Harrison’s Bird Food Adult lifetime super fine maintenance formula for small birds; FL, USA) were always available in the aviary on days where birds did not have training. On days where the birds had training, the bowls containing food pellets were removed in the morning, birds were trained in the afternoon, and the food was returned in the late afternoon once all birds had completed training. This was done so that the birds were motivated by food reward.

### Apparatus

Human participants were seated alone in a room at a desk. Stimuli were presented through Sennheiser HD 201 headphones (Wedemark, Lower Saxony, Germany), and the participants used a mouse to make their responses on an Apple Mini-mac computer (Cupertino, CA, USA) with a 23-inch LG Flatron w2361v screen (55.7 × 39 cm; Seoul, South Korea).

For 40 min each day, each budgerigar was separated from the other birds in a wooden operant box (54.5 length × 39 width × 40.5 height in cm). Birds could respond to visual stimuli on a CarrollTouch infrared touch screen (Elo Touch Solutions, USA). Acoustic stimuli were presented through a Visaton DL 5 8 Ohm speaker (frequency response 150–20,000 Hz; Haan, Germany) that was located directly above the center of the touch screen. The box was lit by an LED houselight that mimicked daylight (6500 Kelvin; Paulman IP67 special line; Vancouver, Canada). Food reward was a highly desirable mix of grains (Versele-Laga Budgies Prestige; Deinze, Belgium) administered to birds through a Campden Instruments 80209 Pellet Dispenser (Loughborough, UK), which was controlled by a Mac mini computer (Cupertino, CA, USA) via an Arduino uno chip (SmartProjects, Italy). The operant box was placed next to the aviary, and an opening in the aviary caging allowed birds to enter and exit the box through a sliding door operated by an experimenter. Each session lasted roughly 40 min. After completing a session, birds were released back into the aviary and the next bird was put into the box.

All stages of training for both humans and budgerigars were programmed in Python using Experimenter (see https://github.com/cogbio/Experimenter).

### Stimuli

Table 1 shows the 24 nonsense words we created, each containing two syllables. The 24 nonsense words were divided into two lists of 12, which were used separately in the experiment. We ensured that these nonsense words did not resemble words in several languages including English, German, French and Italian, by asking native speakers of each language to assess them prior to the experiment. None of these native speakers participated in the actual experiment. Each set of nonsense words contained six syllables in total, and each syllable was used in four nonsense words, twice as the first syllable and twice as the second syllable. One of the sets was used for training and the other for generalization testing, counterbalanced across subjects.

**Table 1:** The two training sets of nonsense words presented to both humans and budgerigars

Training set 1		Training set 2	
Syllable 1	Syllable 2	Syllable 1	Syllable 2
pu	vo	to	su
pu	ga	to	mi
zi	pu	ji	to
ga	pu	su	de
na	ke	de	ji
na	vo	de	lu
ke	na	ji	mi
ga	zi	lu	to
zi	ga	su	lu
ke	zi	mi	ji
vo	na	lu	su
vo	ke	mi	de

Each nonsense word was made up of two syllables and each syllable was only used in one of the two training sets, not both, and each syllable occurred as the first stimulus in two nonsense words, and as the second stimulus in two nonsense words

Prior to running the current experiment, we piloted the experiment extensively with humans. During piloting, we created a set of stimuli consisting of naturally recorded speech. We had 4 native North American English speakers (2 males and 2 females) read the nonsense words both with initial stress and with final stress, and we had participants discriminate the two and then tested them with artificial manipulations of these stimuli using the same design we report below. Afterward, we moved to the entirely artificially manipulated stimuli described below. The reason for this was threefold. (1) Human participants had difficulty learning to identify stress using the nonsense words from natural speech recordings. (2) The artificial stimuli were much more controlled. (3) The significance patterns between the results of the natural speech task and the artificial speech task were the same, so they appeared to be analogous. Accordingly, we report only the results for the artificial stimuli here.

**Fig. 1:**
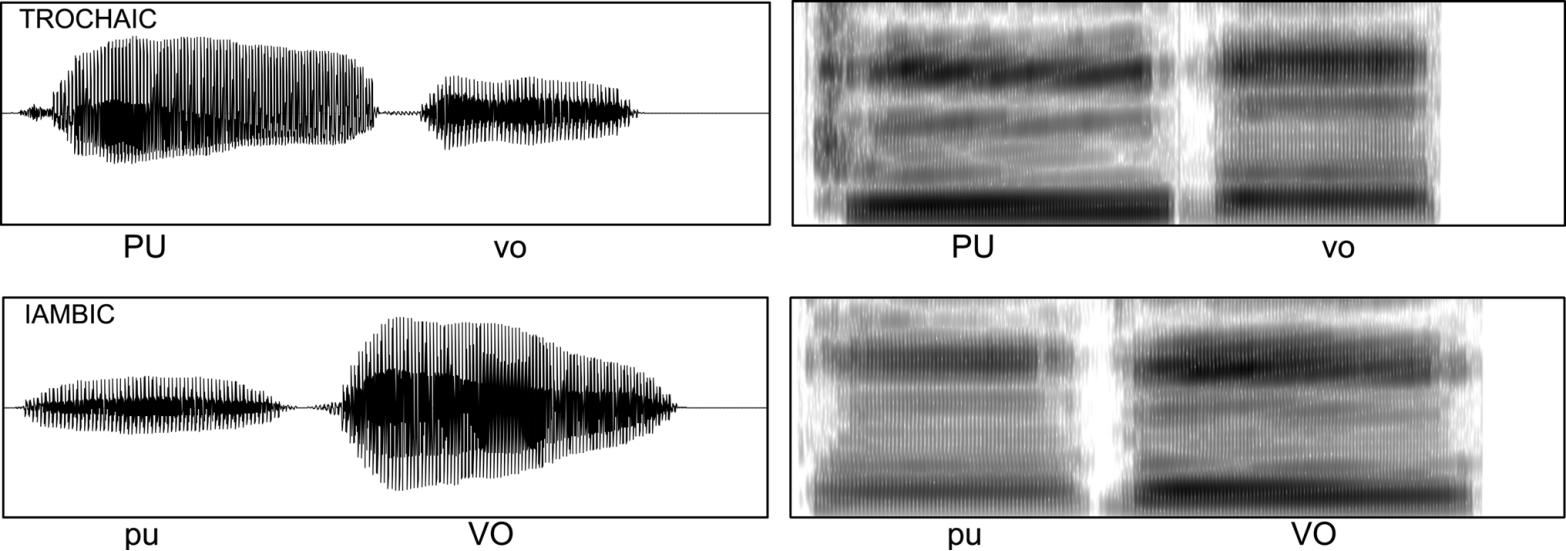
Oscillograms and spectrograms of the trochaic and iambic versions of one of the stimuli (puvo). Both the oscillograms and spectrograms were generated in Praat (see http://www.fon.hum.uva.nl/praat/) and are shown in a 1-s time window (*x* axis). The oscillograms display between −0.4 and +0.4 volts and the spectrograms display frequencies from 0 to 5000 Hz (*y* axis)

To create the artificially manipulated stimuli used in the current experiment, M.H. recorded herself speaking each syllable in a flat tone and the syllables were manipulated in Praat (acoustic synthesis and analysis software, see http://www.fon.hum.uva.nl/praat/) to simulate stressed and unstressed speech. Four features were manipulated to simulate lexical stress: vowel quality, pitch, loudness and duration. Variations in vowel quality were performed during the recording process: M.H. recorded each syllable twice: one with a long vowel sound for the stressed syllables and one with a short vowel sound for the unstressed syllables (e.g., schwa, see Table 2 for how each vowel was pronounced depending on stress). For pitch, unstressed vowels were always 194 Hz, and stressed syllables began at 194 and rose linearly to a peak between 230 and 280 Hz (randomly generated for each syllable). For amplitude, the stressed stimulus was always the same loudness [root mean square amplitude (RMS) of 0.1] and the unstressed stimulus was between 7 and 10 dB quieter (RMS of 0.0316 and 0.0447, randomly generated for each syllable). For duration, the stressed stimulus was always 0.5 s and the unstressed stimulus was always between 0.3 and 0.4 s in length (randomly generated for each syllable). The relative magnitudes of these acoustic differences between stressed and unstressed syllables were based on Fry (1955, 1958). All syllables were combined into the two-syllable nonsense words from Table 1 such that there were no silences between syllables within each stimulus. See Fig. 1 for example oscillograms and spectrograms of the stimuli. The stimuli themselves are available as supplementary material.

**Table 2:** Pronunciation of vowel types for stressed versus unstressed syllables

Vowel	Stressed pronunciation	Unstressed pronunciation
A	[ɔ]	[ə]
E	[eɪ]	[ε]
I	[i:]	[ɪ]
O	[oʊ]	[ə]
U	[u:]	[ə]

**Fig. 2:**
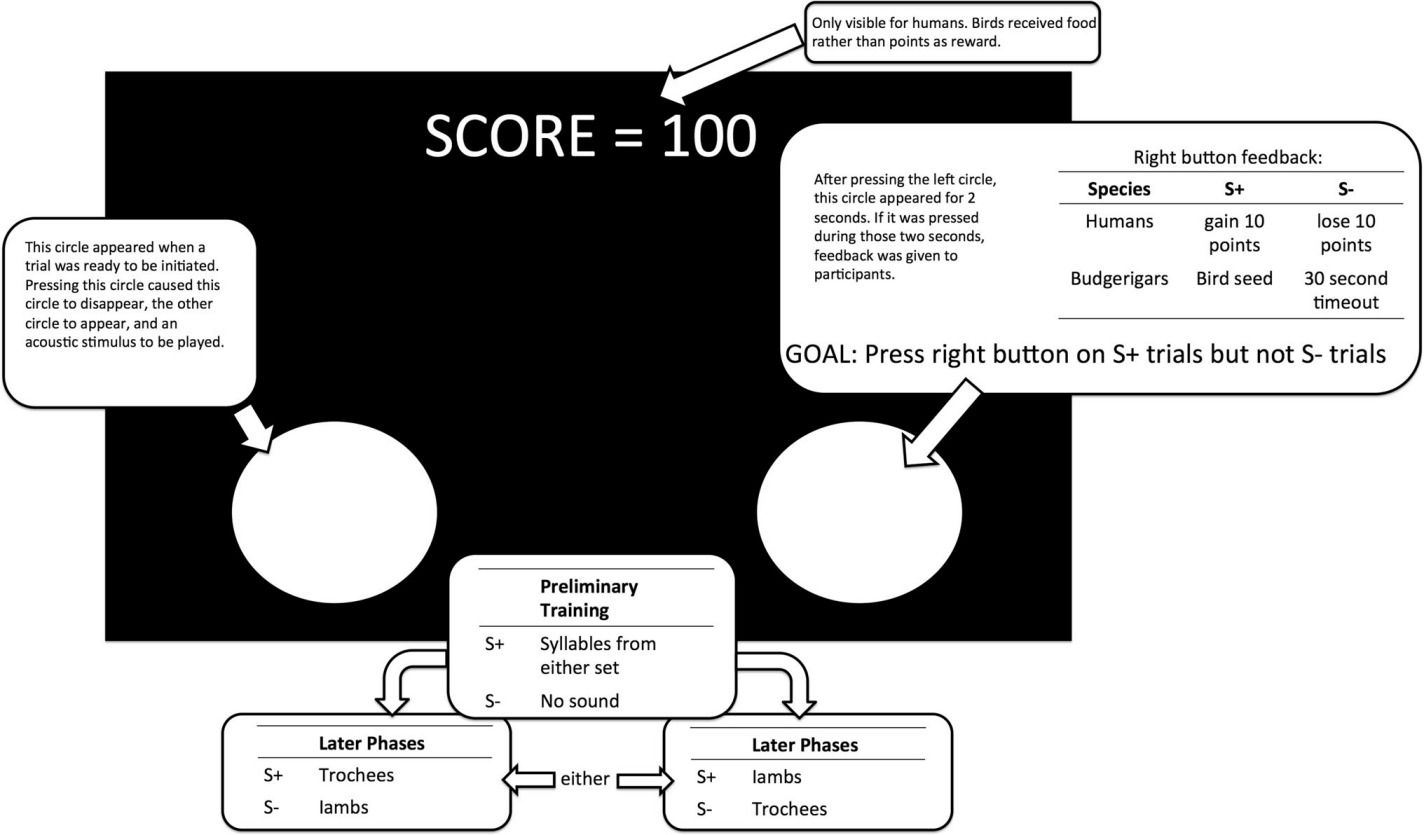
Diagram explaining what the humans and budgerigars experienced on screen during each phase of the experiment

### Procedure

Our overall procedure and sequence of training and test phases were designed to be as similar as reasonably possible for humans and birds. Briefly, we first provided a training phase where participants learned the test system and procedure (e.g., to select an onscreen button to gain a reward), then learned to discriminate between our trochaic and iambic two-syllable nonsense words. After these initial stages, successful participants moved to a test stage including unfamiliar “probe” stimuli (which were not rewarded to avoid further training in this stage). We first examined whether participants generalized to novel stimuli (providing evidence that they had learned a more abstract stress rule, rather than simply memorizing exemplars); after this we presented novel stimuli where various acoustic cues from the training stimuli were omitted (to probe the cues that participants used to assess the stress rule). We now describe these stages in detail.

Prior to preliminary training, human participants were asked to read and sign a consent form. They were informed that their participation was voluntary, and they could stop at any time. They were told that their goal in the experiment was to gain as many points as possible by using a mouse at a computer while listening to sounds via headphones. Participants were not given any instructions on how to achieve points. In fact, they were not given any information on the nature of the acoustic stimuli, or that they were to complete an auditory categorization task. They were shown how to adjust the sound volume to their comfort level at any time during the experiment. Any technical questions were answered (e.g., adjusting headphones), but questions relating to the nature of the experiment were not answered until after completion of the experiment. Instead, participants were prompted to just try their best. This lack of instruction was designed for comparability with the birds, who would otherwise be at a disadvantage.

Budgerigars were trained for 40 min sessions each day. Before starting the experiment, they underwent a visual shape training procedure prior to preliminary training in order to become accustomed to making responses on the touch screen for food reward. This procedure contained 3 phases: (1) Millet image autoshape: Budgerigars were presented with a circle containing an image of millet (a preferred food item) randomly on either the left or right side of the screen. If they touched this image or waited for 30 s for a trial to time out, they received food reward. (2) Arbitrary button autoshape: The circle was now purely white, but otherwise this phase functioned the same way as the first phase. (3) Two button training: The white circle first appeared on the left side of the screen, and, once touched, appeared on the right side of the screen. Budgerigars then had 2 s to respond to the white circle again in order to obtain food reward. Timeouts were not rewarded in phase 3. For each of the 3 shape phases, after budgerigars had completed at least 40 trials (excluding trials with timeouts) for at least 2 sessions they were moved to the next phase. Auditory stimuli during the rest of the experiment were presented at an overall amplitude of approximately 75 dB SPL at the approximate position of the budgerigar’s head.

During the actual experiment, the visual stimuli on screen were the same for both humans and budgerigars, except that the humans had a score bar at the top of the screen that displayed their current score, and received visual feedback of their score increasing or decreasing. We designed this task to be performed on a computer screen so that we could easily perform similar procedures with visual stimuli in further experiments (see “Discussion”). In all stages, participants pressed a circle that appeared on the left side of the screen to initiate a trial. They were then presented with an acoustic stimulus, and were given 2 s where they could respond to the stimulus by pressing a circle that appeared on the right side of the screen. For some acoustic events, pressing the right circle was rewarded (S+) and for other acoustic events, pressing the right circle was unrewarded (S−). Perfect performance for all stages was to respond to rewarded (S+) trials but not to unrewarded (S−) trials. A diagram of the screen with a summary of this information is provided in Fig. 2.

The following sections explain in more detail the training procedures used for both humans and budgerigars:

#### Preliminary training

During preliminary training, at the beginning of a trial, a white circle appeared on screen. Once human participants clicked or budgerigars touched the white circle, it disappeared and another white circle appeared in a location to the right of where the first circle had been. When the right circle appeared, on 50 % of the trials, a sound played. Participants then had 2 s during which they could respond by clicking or touching the right circle. If participants clicked or touched the right circle after a sound (S+), a positive acoustic feedback tone played from the speaker (roughly 600 Hz), and subjects were rewarded either by receiving 10 points added to their visible score (in the case of the humans) or a food reward (in the case of the budgerigars), and after 1 s the left circle reappeared so that they could start a new trial. If participants clicked or touched the right circle after no sound had played (S−), a negative acoustic feedback sound played from the speaker (roughly 200 Hz), and a red screen appeared. Humans also lost 10 points from their score and were given a 5 s delay, and budgerigars did not receive reward and received a 30 s delay before being able to start the next trial. No feedback was given if participants did not respond within the 2 s window. This stage was conducted so that the participants learned the relevance of attending to the acoustic stimuli in order to solve the task.

The sounds played during this stage were single syllables that would be used in pairs in the two-syllable nonsense words in later stages. Both stressed and unstressed syllables were presented. Syllables were presented in a random order without replacement until all syllables had been presented, after which all syllables were randomized again and the procedure was repeated. Each block of 10 trials contained 5 sound and 5 no-sound trials. The criterion to complete this stage for humans was to complete a minimum of 10 trials with an overall discrimination ratio (DR; see “Response measures”) of 0.8 or higher. The maximum number of trials humans could complete at this stage was 100. Budgerigars completed as many trials as they wanted within each 40 min training session. Once budgerigars completed 2 sessions with DR ≥ 0.8, they were moved on to the next phase.

#### Discrimination training

Discrimination training functioned the same way as preliminary training, except for the acoustic stimuli presented. Participants were still required to press the left circle to initiate a sound and then choose whether or not to respond to the sound by pressing the right circle. Now, however, all trials contained two-syllable nonsense words. Trochees (first syllable stressed) were presented on 50 % of the trials and iambs (second syllable stressed) on the other 50 % of the trials. Because we had two training sets containing different syllable types (e.g., “pu” vs “ji”), approximately half of all participants (half of the humans and half of the budgerigars) were trained with each set. Table 1 describes these two sets in more detail. In addition, approximately half of the participants within a training set were rewarded for responding to trochees (S+) and not for responding to iambs (S−), and the other half of participants were rewarded for responding to iambs (S+) and not for responding to trochees (S−). Human participants completed this phase by completing at least 10 trials with an overall DR ≥ 0.8. The maximum number of trials they could complete at this stage was 150. Budgerigars were required to have 4 consecutive daily sessions with a DR ≥ 0.8. Participants were always rewarded at this stage for responding to the correct stress type (e.g., iambs).

#### Pre-testing

Before participants were tested, they completed a pre-testing phase, which functioned the same as discrimination training, except that reinforcement on S+ trials (e.g., trochees) only occurred 85 % of the time instead of 100 % of the time. This was conducted to blur the distinction in outcome to responding to future unrewarded probe trials and training stimuli. Perfect performance at this stage was identical to that of discrimination training (e.g., respond by pressing the right circle to all trochees and not to any iambs). Human participants completed this phase by completing at least 10 trials with an overall DR ≥ 0.8. The maximum number of trials they could complete at this stage was 50. Budgerigars were required to complete at least 3 sessions at this stage with a DR ≥ 0.8 for at least the last 2 sessions.

#### Generalization testing to novel stimuli

This stage followed the same format as pre-testing, except that now unrewarded “probe” trials were added. These probe trials contained novel stimuli that the participants had never heard and were presented to assess whether the participants had simply memorized the training stimuli, or instead had learned a more abstract rule which they could apply to novel stimuli. These stimuli were not rewarded or punished so as not to influence responding to future probe stimuli (e.g., if a novel stimulus is rewarded it might influence participants to respond to all novel stimuli). Unrewarded probe stimuli were presented on 20 % of trials (2 trials in each block of 10 trials were probe trials, of the remaining 8 trials, 4 were trained S+ and 4 were trained S− trials) and consisted of stimuli from the alternate training set (e.g., “jito” instead of “puvo”) that a participant had not been trained with (i.e., if a participant was trained with set 1, they were probe tested with set 2 and vice versa). Each participant heard each stimulus from the other training set once for a total of 120 trials (24 probe stimuli + 4× each of the 24 training stimuli). Once a participant had completed all 120 trials, they returned to the pre-testing phase in preparation for the next phase. To complete the second pre-testing phase, human participants were required to complete at least 10 trials with an overall DR ≥ 0.8. Budgerigars were required to have 1 session with a DR ≥ 0.8 before moving on to the second generalization test. This was done to ensure that discrimination levels remained high before participants completed the second test.

#### Generalization testing to stimuli with absent cues

Generalization testing followed the same format as the novel stimuli test, except instead of presenting stimuli from the other training set during unrewarded probe trials, stimuli were presented with some cues absent. The unrewarded probes with absent cues were alterations of the stimulus set that had been used during training. The difference between these unrewarded probes and the training stimuli was that they contained either only one or all but one of the four cues of stress (pitch, duration, amplitude, and vowel quality). We thus had a total of 8 probe stimulus categories for this stage: pitch removed, duration removed, amplitude removed and vowel quality removed, and also pitch only, duration only, amplitude only vowel quality only stimuli. For absent cues, stressed and unstressed syllables had the same values. To remove vowel quality as a cue, we used the stressed vowel quality for both the stressed and unstressed syllable (see Table 2). To remove pitch as a cue, we used the unstressed flat pitch contour (194 Hz) for both the stressed and unstressed syllable. To remove amplitude as a cue, we used the stressed amplitude (RMS of 0.1) for both the stressed and unstressed syllable. To remove duration as a cue, we used the stressed syllable length (0.5 s) for both the stressed and unstressed syllable. We created all 8 probe stimulus categories (vowel quality removed, pitch removed, amplitude removed, duration removed, vowel quality only, pitch only, amplitude only, duration only) for each training stimulus, and used a random subset [48 stimuli: 3 random exemplars for each of 8 manipulations for each stimulus type (i.e., trochaic or iambic)] to test each participant. As in the first test, 20 % of trials were unrewarded probe trials (2 trials in each block of 10 trials were probe trials, 4 were S+ and 4 were S−) resulting in a total of 240 trials (48 probe trials + 4 × 48 training trials). Once a participant had completed all 240 trials, they had completed the experiment. Although most completed in 1 session, for one of the budgerigars, this test was completed over two sessions because the budgerigar lost interest before completing all 240 trials.

#### Experiment completion

Upon completing the experiment, human participants filled out a form about asking them about their language and musical background and to describe the strategy they used to complete the task. They were then given a debriefing form explaining the goals of the experiment, and any questions they had were answered. All forms were provided in English to accommodate participants with imperfect command of German (e.g., non-local students), but participants were given the option to answer the survey in German if they preferred. Human participants were given 10 € as compensation for their participation.

#### Response measures

To determine whether the humans and budgerigars had successfully learned to discriminate among the nonsense words, we calculated a discrimination ratio (DR) between the S+ and S− stimuli. To calculate the DRs, we divided the percent response for the S+ stimuli by the sum of the percent response for the S+ stimuli and the S− stimuli:

$${\text{DR}} = [\% {\text{ response to }}S + \left] {/(} \right[\% {\text{ response to }}S + ] + \left[ {\% {\text{ response to }}S - } \right])$$

A DR of 0.5 indicates equal responding to both S+ and S−, while a higher DR means more responding to S+ and a lower DR means more responding to S−.

## Results

### Discrimination

Not all participants learned the task. For the humans, 21/31 participants achieved a DR of 0.8 within 150 trials or less of discrimination training. Participants took between 13 and 131 trials to reach this criterion. One of these participants subsequently failed to achieve a DR of 0.8 or higher during the maximum 50 trials of pre-testing and was removed from the analysis, thus 20/31 humans were included in the final analysis.

For the budgerigars, 3/6 birds achieved a DR of 0.8, taking between 49 and 92 sessions each to learn the task. Each session had a variable number of trials (*M* = 103). The other 3 birds were all run for >130 sessions but did not reach criterion. Of the participants who were successful, 11/20 humans and 1/3 budgerigars were trained to respond to trochees (9/20 and 2/3, respectively, were trained to respond to iambs). In addition, 10/20 humans and 1/3 budgerigars were trained with set 1 (10/20 and 2/3, respectively, were trained with set 2).

For the humans, we collected language and musical history data. In total, 14 of the 31 participants reported having had musical training. A *χ*^2^ test showed that there was no difference between participants with musical training and those without musical training in terms of whether or not they solved the task (*χ*^2^ = 2.20, *P* = 0.134).

Additionally, 20 participants were native German speakers. The other 11 participants had 10 different first languages: Slovak, English, Urdu, Persian, Hindi, Malayalam, Bosnian, Chinese, Spanish and Albanian. A *χ*^2^ test showed that there was also no difference between participants who were native German speakers and those who learned another language first in terms of whether or not they solved the task (*χ*^2^ = 2.71, *P* = 0.106).

### Generalization to novel stimuli

**Fig. 3:**
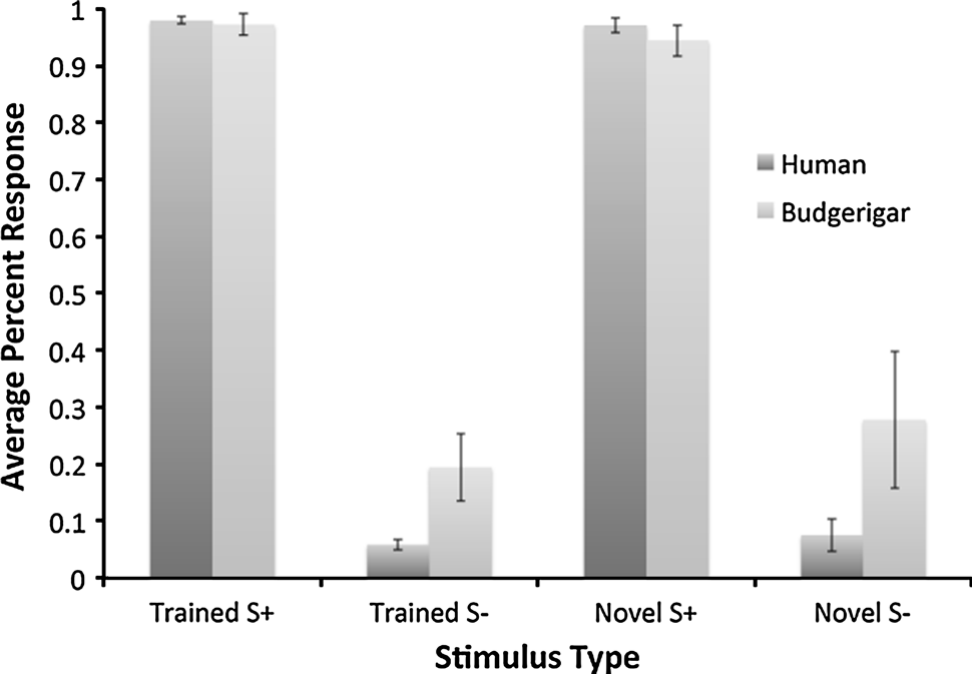
Average percent response to trained S+ and trained S− stimuli as well as novel S+ category and novel S− category stimuli during the generalization test for each species. Note that responses to the novel stimuli resulted in no feedback, but the trained stimuli continued to be reinforced the same way as during pretesting. Error bars show standard error of the mean

For the participants that learned the task, we could evaluate whether they generalized what they had learned to novel stimuli. Figure 3 shows the average percent response to trained and novel S+ and S− nonsense words for each species during the generalization test. We used binomial tests for dichotomous data for each species to determine whether the number of responses that were directed to the trained rewarded stimulus category (e.g., trochaic) were greater than the number of responses that were directed to the trained unrewarded stimulus category (e.g., iambic). We found that both humans (*z* = 43.00, *P* < 0.001) and budgerigars (*z* = 6.93, *P* < 0.001) generalized successfully to novel stimuli by displaying significantly more correct responses than expected by chance (50 %). Because each individual completed 24 trials with the novel nonsense words, we also looked at whether individuals had more correct responses than expected by chance. We found that all humans participants that learned the task generalized (all *z*s ≥ 2.45, all *P*s ≤ 0.014) and two (both *zs* ≥ 4.00, both *Ps* < 0.001) of the three budgerigars generalized and the third approached significance (*z* = 1.89, *P* = 0.059).

### Generalization to stimuli with absent cues

**Fig. 4:**
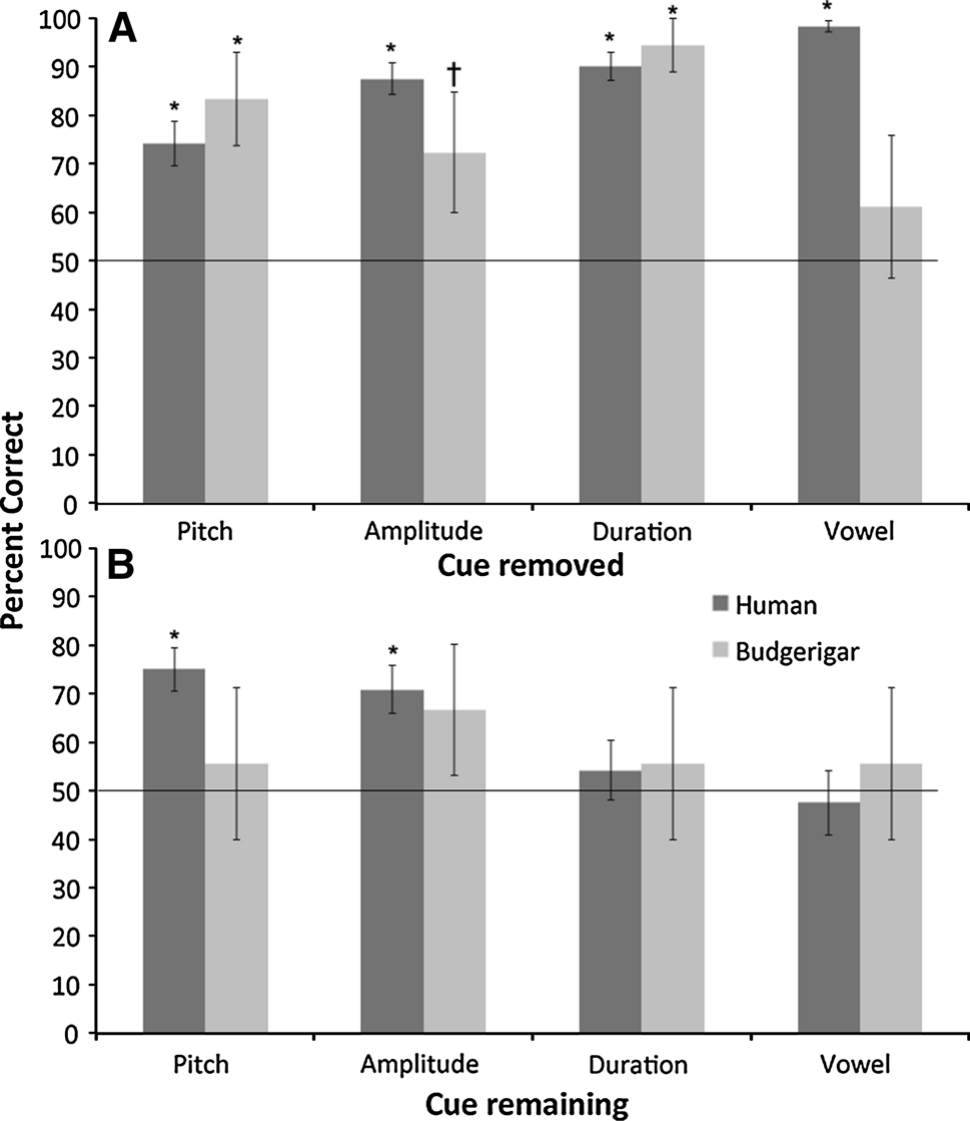
Percent correct for each manipulated stimulus category for each species. Panel **a** shows the manipulated stimuli with one cue removed. Panel **b** shows the manipulated stimuli with only one cue remaining. The line across each graph represents chance. *Error bars* show standard error of the mean. *Stars* show bars that are significantly above chance. *Two-tailed significance, ^†^one-tailed significance

We also evaluated how participants that learned the task responded to training stimuli with absent cues (see Fig. 4). We used binomial tests for dichotomous data for each species to determine whether the number of responses that were directed to the trained rewarded stimulus category (e.g., trochaic) were greater than the number of responses that were directed to the trained unrewarded stimulus category (e.g., iambic) for each type of stimulus with absent cues (pitch removed, duration removed, amplitude removed, vowel quality removed, pitch only, duration only, amplitude only, vowel quality only). Here, there were some differences among the species. Humans successfully maintained discrimination for all stimuli with absent cues (*z* ≥ 4.23, *P* < 0.001) except vowel only (*z* = −0.37, *P* = 0.711) and duration only (*z* = 0.67, *P* < 0.503) stimuli. Budgerigars, however, did not generalize to any of the stimuli with only one available cue (*z*s ≤ 1.22, *P* ≥ 0.222). They did, however, generalize to stimuli with only duration removed (*z* = 8.00, *P* < 0.001), and pitch removed (*z* = 3.46, *P* < 0.001), but not vowel quality removed (*z* = 0.76, *P* = 0.448). Generalization to stimuli with amplitude removed approached significance (*z* = 1.79, *P* = 0.073). Here, unfortunately we could not look at individual data because of the low number of trials with each probe type for each individual.

## Discussion

These results show that both humans and budgerigars can successfully learn to discriminate between trochaic and iambic nonsense words and then generalize to novel stimuli using closely matched methods. Our test results for responses to stimuli with absent cues suggest that humans had trouble identifying the stress pattern if only duration or only vowel quality were available as cues, which suggests humans were attending primarily to amplitude and pitch. Budgerigars had difficulty if more than one cue was absent, probably because the stimuli sounded very different from training stimuli; however, they were able to solve the task without duration or without pitch as a cue, which implies that duration and pitch were not necessary for them to solve the task.

Our results with the budgerigars add to the small but growing area of research studying prosody in non-human animals. Initial studies showed that cotton-top tamarin monkeys (*Saguinus oedipus*), rats and java sparrows attend to prosodic cues when discriminating human languages (Ramus et al. 2000; Toro et al. 2003; Naoi et al. 2012). More recent work has focused on what biases animals have when encountering strings of sounds. For example, de la Mora et al. (2013) showed that rats, like humans, have a bias toward grouping continuous streams of alternating elements as trochaic if the elements alternate in pitch or intensity. Spierings and ten Cate (2014) showed that zebra finches trained to discriminate strings of sounds that differ both in syntax and prosody preferentially attend to prosody. Our study shows that budgerigars can discriminate 2-element strings that differ only in prosodic cues. Taken together, these results suggest that the capacity to attend to prosodic patterns can be found in many animals other than humans.

The human results are not surprising. We know that humans can readily attend to lexical stress if their native language makes use of it (Yu and Andruski 2010; Dupoux et al. 1997), such languages are known as “stress-timed languages” and include English, Dutch and German (Grabe and Low 2002). Given that the majority of our participants were German native speakers, it also makes sense that the participants depended on pitch, as pitch is thought to be the primary signal of lexical stress in many stress-timed languages including German (Kohler 2012). In many of the studies in the literature, human participants are overtly asked to indicate where they hear stress (e.g., Fry 1958; Yu and Andruski 2010; Kohler 2012). Our similar findings here suggest that the go/no-go paradigm with artificial nonsense words taps into the same mechanisms. It would be interesting to see whether cultures that depend more on other features (e.g., duration for Estonians; Lehiste and Fox 1992) might show a response pattern favoring other cues (e.g., duration) in our task.

Examining cultural/linguistic differences in our task could lead to a clearer comparison with the budgerigars. In our task, the budgerigars appeared to be least impaired when identifying stress patterns if pitch and duration were removed. Humans, in contrast, had trouble only when both amplitude and pitch were missing. This suggests that, when identifying stress in human vocalizations, the role of pitch may be greater for humans than budgerigars. However, it is difficult to generalize across all humans given the heavy reliance on pitch by German speakers (Kohler 2012). Interestingly, German speakers can focus on vowel quality if pitch is removed as a cue (Kohler 2012), and, in a recent study with zebra finches, the finches also focused on pitch when determining stress, but they did not appear to have vowel quality available as a perceptual cue (Spierlings and ten Cate 2014). Because there are variations among human populations in terms of which acoustic features they rely on to determine lexical stress, it is also unclear how much our results with the budgerigars are dependent on the experiences and dialects of our budgerigar colony and whether a different colony might favor different features. Thus, it is unclear whether humans and budgerigars would rely on the same cues under slightly different circumstances. Nevertheless, studying cultural variation in performance of our task could help us determine whether any differences we see among species derive from biological constraints. In addition, studies of additional species and comparing the relevant cues within their natural vocalizations and the ones used in the perception of stress categories may shed further light on this topic.

Taking a step back, given the complexity of this task, the fact that budgerigars even solved the task and generalized the rule to novel exemplars is remarkable. Not only did the task require forming categories of “stressed” and “unstressed” syllables, but it required learning that the order of these categories within a continuous speech stimulus determined whether or not a stimulus was reinforced. Thus, ours was a more abstract task than a simple perceptual task such as if we had trained the birds simply to discriminate stressed from unstressed syllables. Importantly, despite lexical stress being a pervasive human linguistic phenomenon, solving the task was not easy for the humans. In fact, only about two-thirds of our human participants successfully learned our task. This clearly underlines how important it is to use comparable methods between humans and non-human animals before drawing conclusions concerning what non-human species can and cannot do.

We used human speech stimuli for this task, but it is unclear whether that was important to obtain the current results. Ultimately, what we showed here is pattern learning (i.e., respond to AB but not BA). What if we had used a different set of stimuli such as budgerigar vocal stimuli, or musical stimuli, or even arbitrary sounds? If a given species or human cultural group uses the same cues regardless of the type of task, then it would suggest pattern detection across domains is influenced by the same perceptual processes.

While language-like stimuli are more biologically relevant for humans than other species, studying these same patterns using different elements across species can help to disentangle what aspects of our perception of language are rooted in more basic acoustic perception. It is possible that these acoustic perceptual abilities go well beyond just vocalizations. In humans an obvious example of acoustic grouping that does not involve vocalizations is instrumental music, but more generally, the non-vocal sounds created by animal movements can often contain acoustic patterns. For example, wing flapping in birds, which contains biologically relevant information for our budgerigars, contain alternating up/down acoustic elements, much like the stimuli used in our study. Larsson (2012, 2014, 2015) has suggested that perception and evaluation of such locomotor acoustic patterns may have been important in the development of acoustic learning abilities. Thus, acoustic grouping of patterns like the ones used in our study may be an underlying ability in many species because of their evolutionary history.

However, in humans it appears that experience with vocalizations has a strong influence on grouping ability. For example, we know from human data on acoustic grouping that some experience with certain languages is necessary for grouping based on duration (Iversen et al. 2008; Bion et al. 2011), but not based on intensity (Hay and Diehl 2007; Iversen et al. 2008). As our budgerigars also did not rely on duration, but appeared to rely to some degree on amplitude, our data are consistent with these findings. A relevant follow-up might thus investigate under what conditions a non-human animal develops grouping biases based on duration.

It is not just acoustic grouping that may be relevant here. In fact, as we mentioned earlier, recent research has shown that visual parallels of the iambic–trochaic law seems to be present in humans (Peña et al. 2011). Specifically, the authors found that visual stimuli that differed in intensity were grouped as trochaic, and visual stimuli that differed in length were grouped as iambic. It would be interesting to study whether there are similar parallels across species. Are species that can solve our acoustic task more likely to be able to solve an analogous visual task and vice versa? In addition, would their response patterns be parallel across domains? Given the touch screen apparatus we used in the present study, we could easily conduct a visual version of this study with only subtle changes to the methods. This is a research direction that we are currently pursuing.

In conclusion, our results support the idea that the mechanisms underlying the processing of metrical stress by humans are present in at least one other species. From here there are several questions that we plan to address in follow-up studies: (1) Do our human results hold cross-culturally? (2) What other species can solve this kind of task (e.g., vocal learners, social animals, animals that move in groups, etc.)? (3) Are these results domain-specific? (4) What kind of potentially more general underlying grouping mechanisms might explain the ability to detect acoustic stress across species? (5) What is the most optimal stimulus to enhance perception of lexical stress and are there limitations on stimulus features (e.g., a critical time gap between sound A and B)?

## Electronic supplementary material

Below is the link to the electronic supplementary material.

Supplementary material 1 (ZIP 28778 kb)
